# Evaluation for Bearing Wear States Based on Online Oil Multi-Parameters Monitoring

**DOI:** 10.3390/s18041111

**Published:** 2018-04-05

**Authors:** Si-Yuan Wang, Ding-Xin Yang, Hai-Feng Hu

**Affiliations:** Science and Technology on Integrated Logistics Support Laboratory, National University of Defense Technology, Changsha 410073, China; 13272090980@163.com (S.-Y.W.); hhf_online@163.com (H.-F.H.)

**Keywords:** online oil monitoring, bearing state evaluation, multiple sensors

## Abstract

As bearings are critical components of a mechanical system, it is important to characterize their wear states and evaluate health conditions. In this paper, a novel approach for analyzing the relationship between online oil multi-parameter monitoring samples and bearing wear states has been proposed based on an improved gray k-means clustering model (G-KCM). First, an online monitoring system with multiple sensors for bearings is established, obtaining oil multi-parameter data and vibration signals for bearings through the whole lifetime. Secondly, a gray correlation degree distance matrix is generated using a gray correlation model (GCM) to express the relationship of oil monitoring samples at different times and then a KCM is applied to cluster the matrix. Analysis and experimental results show that there is an obvious correspondence that state changing coincides basically in time between the lubricants’ multi-parameters and the bearings’ wear states. It also has shown that online oil samples with multi-parameters have early wear failure prediction ability for bearings superior to vibration signals. It is expected to realize online oil monitoring and evaluation for bearing health condition and to provide a novel approach for early identification of bearing-related failure modes.

## 1. Introduction

The engine is regarded as the heart of the vehicle, and its bearings’ working status is directly related to the stability. To prevent failure, especially in harsh environments or critical moments, it is of great significance to monitor and evaluate the health state of bearings since their failure in general can cause injuries and financial losses when faults are poorly diagnosed. The early fault diagnosis of bearings has attracted lots of attention [1]. Vibration signals processing and lubrication oil analysis are the most often adopted methods to fulfill the task [2,3,4,5]. Acoustic analysis with classification can also be used for fault diagnosis of rolling bearings as presented in [6]. However, there is some lack of knowledge about the fault diagnosis method based on vibration or acoustic signals analysis for bearings in terms of high frequency of compound faults [7] and mechanical systems vibrating intensively with large noise. Oil parameters are capable of reflecting the abrasion of the contact surfaces of shaft, bearing, gear, and cam during operation. The information of different contaminant particles and wear debris can provide scientific and reasonable basis for the equipment state assessment [8]. Currently, the oil analysis methods mainly include offline and online approaches. The offline approach includes the analysis of IR spectrum, ferrography analysis, and so on, which are generally effective techniques to assess bearing wear states [9,10,11,12,13]. Jingwei Gao, et al. [14] used a proportional model to find the abnormal wear information in spectrum data, give more accurate results of wear condition, and give the fault modes in the engine. However, on the one hand, the iron spectrum is not very sensitive to non-ferromagnetic particles. On the other hand, this method is hard to reflect promptly the wear states of mechanical parts due to the long oil sampling interval. Additionally, the process of obtaining oil samples is too tedious, what with tracking the time of filling and changing oil in a batch of engines in the long run. It is so prone to inducing detection error which has a great impact on the accuracy of wear state evaluation of mechanical parts.

The lubricating oil is just like the “blood” of the engine, and its online monitoring has been a research focus in recent years. Wear debris analysis is becoming the most powerful oil analysis strategy for assessing machine condition [15]. The BBRB (a multilevel belief rule base) system [16] was proposed for wear mode identification in real-world diesel engines with better performance and stability than competing systems. T. H. Loutas et al. [17] fused three techniques of online monitoring including oil, acoustic wave, and vibration signals to detect the health condition of machines. A first testing approach using a highly accelerated life test chamber was presented to assess the performance of oil properties sensors under extreme ambient temperature and vibration levels, based on measurements from a wind turbine gearbox in [18]. Tao Hui and Shizhong He [19] carried out online oil monitoring on engines of the offshore drilling platform based on the changing trends of large grain wear. Xiaoliang Zhu et al. [20] provided a comprehensive review of the state-of-the-art online sensors for measurement of the oil properties with a particular focus on the developments in the past few years. However, because of the complex information and different impact factors on oil parameters, there are many different appearances on the same fault, and one feature may be caused by a number of failures. In other words, there is a typical nonlinear mapping between fault and its feature information. Therefore, it is difficult for a single oil parameter to evaluate effectively the health condition of a machine. Huiliang Gao et al. [21] have verified that the multiple sensors evaluation results are more reliable than online monitoring systems with a single sensor. Zhixiong Li and Xinping Yan [22] used an intelligent data fusion method based on principal component analysis (PCA) and a genetic algorithm fuzzy neural network (GAFNN) to analyze oil chemical compositions and microscopic debris for identifying the rolling bearing conditions, and also verified that the sensor fusion technique produces superior results to those using single measurements. Although much research has been done on bearing online monitoring based on oil multi-parameters, little work has been done on which parameter of oil is critical and which one is insignificant. To the authors’ knowledge, little is known about whether it is accurate to use multiple oil parameters to evaluate bearing health conditions online. These are the motivations for this study.

To sum up, it is of great importance to establish a new and accurate evaluation scheme based on oil lubricating multi-parameters and explore a correspondence between oil parameters features and the changing of bearing wear states. Based on the above background research, we studied the technique of online oil monitoring and evaluation for bearing wear states based on lubrication multi-parameters obtained through the online oil multi-parameters monitoring system (OOMS). Through monitoring oil multi-parameters by multiple sensors, we aimed at obtaining the characteristics comprising oil quality indications, oil metal debris, pollutants, and so on. Furthermore, the wear states of bearings in this system could be evaluated by analyzing these characteristics and their changing trends. We proposed a novel model optimized k-means clustering model to evaluate bearing wear states. The specific research and implementation route are as follows. A testing platform was built for carrying out the bearings’ whole life test. During the experiment, oil multi-parameters such as micro-metal debris, pollution, viscosity, water, density indications, and vibration signals were monitored and acquired. Then, an approach for analyzing the relationship between oil parameters and wear states was proposed based on an improved k-means clustering model (KCM) optimized by a gray correlation degree model (GCM). First, a correlation degree distance matrix was generated using GCM to express the relationship of samples of oil multi-parameters of different times. Then the matrix was clustered using KCM and the oil multi-parameter samples were clustered into different clusters. The experimental results show that lubricating oil components contain vital information about the bearing wear states and there is an obvious correlation between the clusters and the bearing wear states. To verify the state evaluation results, bearing states were also identified using vibration signals, which are basically consistent with that of oil multi-parameters. Furthermore, we found that the time points for bearing wear states changing through oil multi-parameters were earlier than the time points obtained through vibration signals, which indicates that lubricating oil multi-parameters have the advantage over vibration signals in bearing early wear failure prediction.

The rest of this article is organized as follows: Section 2 introduces the theory and outline of KCM and GCM. Section 3 shows the experiment procedures and contents. Section 4 shows the experiment results and analysis, then Section 5 discusses the reason for different analytic results between oil parameters and vibration signals. Finally, Section 6 draws the conclusion of this study.

## 2. Bearing Wear States Clustering Model Based on Online Oil Samples of Multi-Parameters

### 2.1. Outline of KCM Algorithm

**Figure 1:**
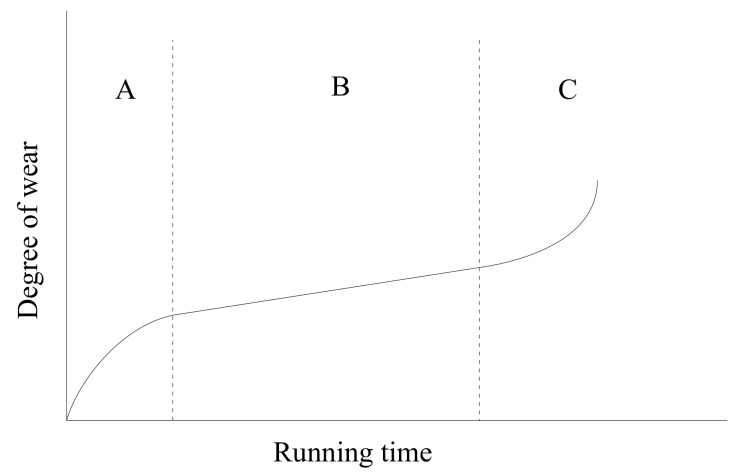
Bearing wear states curve through the whole lifetime.

In general, the wear states curve of bearings through their whole life is shown in Figure 1. It can be roughly divided into three stages: grinding period as A, normal wear period as B, and serious wear period as C.

Due to the fact that the composition of lubricating oil is affected by the bearing wear states, in theory, there could be three different stages for online oil samples which can reflect the different wear states of bearings. Therefore, aimed at the problem about data aggregation or wear modes classification, the appropriate clustering algorithm is considered for solving such problems. If we can obtain online oil monitoring samples of bearings through the whole lifetime, the samples can be classified into three clusters, and each cluster in the time domain would correspond to a different wear stage of bearings.

**Figure 2:**
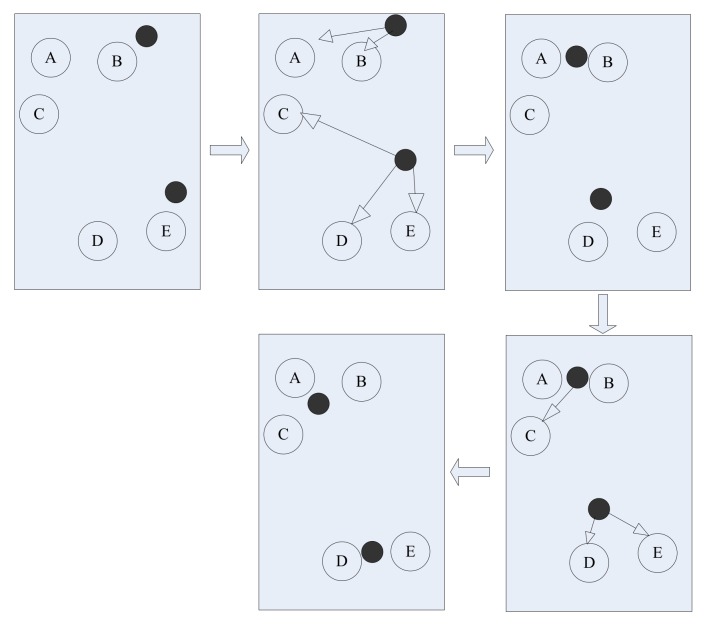
K-means clustering principle diagram.

The k-means clustering model algorithm takes distance as the evaluation indication of similarity, which means that the closer the two objects are, the greater their similarity is. KCM classifies samples close to each other into the same cluster. The schematic diagram of the algorithm is shown in Figure 2. The two black dots are set as two clustering centers in order to divide those circles relatively close to the black dots into two major classes. This algorithm is a traditional method of clustering analysis with the characteristics of high efficiency and less calculation. What’s more, the clustering quality is improved asymptotically. The KCM method is suitable for the clustering of distance in the most classification cases.

As for the deficiency of the KCM method, on the one hand, according to the bearings’ whole life testing, their wear states go through three stages from beginning to failure as shown in Figure 1. Assuming that the test data could be divided into three clusters of the same wear states, then the K value in the k-means algorithm can be set in advance. The lubricating oil parameters contain the bearing wear states information, which can reflect the evolution trends of faults. The specific data of oil parameters are not in the range of k-means algorithm outliers, because the rule of changing trends is hidden in oil samples. At the same time, the benefit of using multiple oil parameters is the ability to prevent one single parameter from outliers that causes an error in the result. The experimental data and analysis below have revealed that there are some interconnections and constraints between the various parameters of oil. Further verification is done by comparing the results of different multi-parameter combinations in Table 7. As a result, the defect of the k-means algorithm that is sensitive to outliers can be avoided and does not have a significant impact on the experimental analysis results. On the other hand, some of the deficiencies of the KCM method need to be made up for using the gray correlation model. For example, the lubrication system should be regarded as a gray system in which some information is visible and the others are unknown, and it is difficult for KCM to obtain desirable results by clustering some unknown data directly. In addition, the deficiency of the KCM algorithm, such as easily trapping in local optimum and generating numerous iterations, can be improved by the gray correlation model. Figures 17a,b and 19a,b show that the clustering result directly by KCM could fail to get any information about bearing status, but G-KCM improves to some extent.

### 2.2. Outline of Gray Correlation Model

Gray system theory proposes a gray correlation degree model to find the correlations between subsystems or sub-factors in the system [23,24]. Before samples clustering, the gray correlation degree of oil samples is calculated by GCM to quantify the correlation between different samples. Based on the gray correlation degree, the correlation matrix is calculated. The matrix is used as input to KCM clustering, which can reduce the iteration number of the k-means algorithm, simplify the complexity of the algorithm, and reduce the number of iterations of the algorithm. At the same time, owing to that each oil sample is used to calculate the correlation degree with all the rest of the data, the defect of KCM falling into local optimum is overcome.

Therefore, after the data is preprocessed, we firstly calculate the correlation degree of the data samples using GCM. Then KCM is used to cluster the obtained correlation matrix. When analyzing the data samples by GCM, the dimensionless processing should be performed for measurement quantities of different dimensions.

Assuming $X_{0}=(x_{0}(1),x_{0}(2),\cdots ,x_{0}(n))$ is the characteristic behavior sequence of the system, and

$$X_{1}=(x_{1}(1),x_{1}(2),\cdots ,x_{1}(n))$$

$$X_{i}=(x_{i}(1),x_{i}(2),\cdots ,x_{i}(n))$$

$$X_{m}=(x_{m}(1),x_{m}(2),\cdots ,x_{m}(n))$$

are the related sequences, in this paper, the data normalization is used as follows:

(1) $$x_{i}^{\prime }(k)=\frac{x_{i}(k)-\underset{i}{\min}x(k)}{\underset{i}{\max}x(k)-\underset{i}{\min}x(k)}$$

The gray correlation coefficient $\gamma (x_{0}(k),x_{i}(k))$ of $x_{0}(k)$ and $x_{i}(k)$ is defined as [25]

(2) $$\gamma (x_{0}(k),x_{i}(k))=\frac{\underset{i}{\min}\underset{k}{\min}\left|x_{0}(k)-x_{i}(k)\right|+\xi \underset{i}{\max}\underset{k}{\max}\left|x_{0}(k)-x_{i}(k)\right|}{\left|x_{0}(k)-x_{i}(k)\right|+\xi \underset{i}{\max}\underset{k}{\max}\left|x_{0}(k)-x_{i}(k)\right|}\begin{array}{cc} , & i=1,2,\cdots m \end{array}$$

Before $\gamma (x_{0}(k),x_{i}(k))$ is calculated, all of the related sequences must be initialized as steps presented in [26]. The gray correlation degree of sequences $X_{0}$ and $X_{i}$, $\gamma (X_{0},X_{i})$ is defined as

(3) $$\gamma (X_{0},X_{i})=\frac{1}{n}\sum_{k=1}^{n}\gamma (x_{0}(k),x_{i}(k))$$

$\gamma (X_{0},X_{i})$ is usually denoted as $\gamma_{0i}$, and $\gamma_{0i}(k)$ denotes the correlation coefficient of $\gamma (x_{0}(k),x_{i}(k))$. ξ is distinguish coefficient, and ξ∈(0,1).

### 2.3. G-KCM Algorithm and Its Implementation

The specific implementation of the gray k-mean clustering algorithm is as follows.

Step 1: Calculate the initial value (or the mean value) of each sequence.

(4) xi′=Xixi(1)=(xi′(1),xi′(2),⋯,xi′(n)),i=0,1,2,⋯,m

Step 2: Obtain the absolute value sequence of the difference between $X_{i}$ and $X_{j}$.

Note $\Delta_{ij}(k)=\left|x_{i}^{\prime }(k)-x_{j}^{\prime }(k)\right|$ and $\Delta_{ij}=(\Delta_{ij}(1),\Delta_{ij}(2),\cdots ,\Delta_{ij}(n))$, j=1,2,⋯,m.

Step 3: Find the maximum and minimum value of $\Delta_{ij}(k)=\left|x_{i}^{\prime }(k)-x_{j}^{\prime }(k)\right|$, k=1,2,⋯,n and j=1,2,⋯,m, which are marked, respectively, as

(5) $$M=\underset{j}{\max}\underset{k}{\max}\Delta_{ij}(k)$$

and

(6) $$m=\underset{j}{\min}\underset{k}{\min}\Delta_{ij}(k)$$

Step 4: Calculate the correlation coefficient according to Equation (2)

(7) $$\gamma_{ij}(k)=\frac{m+\xi M}{\Delta_{ij}(k)+\xi M}$$

where ξ∈(0,1), k=1,2,⋯,n, i=1,2,⋯,m and j=1,2,⋯,m.

Step 5: Calculate the average of $\gamma_{ij}$ according to Equation (3), the gray correlation degree

(8) $$\gamma_{ij}=\frac{1}{n}\sum_{k=1}^{n}\gamma_{ij}(k),i=1,2,\cdots ,m$$

Step 6: Repeat above steps 2–5 to calculate all the gray correlation degrees. And the gray correlation degree matrix R (R is the symmetric matrix) is obtained as

$$R=\left[\begin{array}{llll} \gamma_{11} & \gamma_{12} & \cdots & \gamma_{1m}\\ \gamma_{21} & \gamma_{22} & \cdots & \gamma_{2m}\\ \vdots & \vdots & \ddots & \vdots \\ \gamma_{m1} & \gamma_{m2} & \cdots & \gamma_{mm} \end{array}\right]$$

Step 7: Input the matrix R to KCM. Given k as the number of cluster centers, KCM classifies R into k clusters. The row vector of matrix *R* can be represented as r(γ(1),γ(2),⋯,γ(j)), j=1,2,⋯m.

Step 8: Select the initial center point, like c(1)=γ(1), ⋯, c(k)=γ(k).

As for R, each row vector compares with c(1),c(2),⋯,c(i) in turn, for i=1,2,⋯k, and then the one which has the smallest difference with c(i) is marked as $\gamma_{g_{i}}(j)$ , and $g_{i}$ counting once.

Step 9: Set *m* as the number of $\gamma_{g_{i}}(j)$ marked i, and recalculate

(9) $$c(i)=\frac{1}{m}\sum_{g_{i}=1}^{m}\gamma_{g_{i}}(j),g_{i}=1,2,\cdots m$$

Step 10: Repeat steps 8–9 until all the changes of the c(i) are less than the given threshold, and finally the clustering results are obtained.

## 3. Experiment for Bearing States Evaluation Based on G-KCM

### 3.1. Design of the Scheme for Bearing States Evaluation

In order to realize the bearing wear state evaluation based on online lubricating oil multi-parameters, the whole lifetime test of rolling bearings was performed on a bearing fatigue test machine. During the test, multiple online oil parameters such as metal debris and physical and chemical properties, etc., were recorded using monitoring sensors. The experimental data acquisition system has been built up to collect the multiple oil parameters and vibration signals from new bearings to complete failure states under appropriate loads. The online oil monitoring system is abbreviated as OOMS, and the fatigue experiment of a bearing’s whole life is FEOBL, which provided data support for bearing wear states analysis.

**Figure 3:**
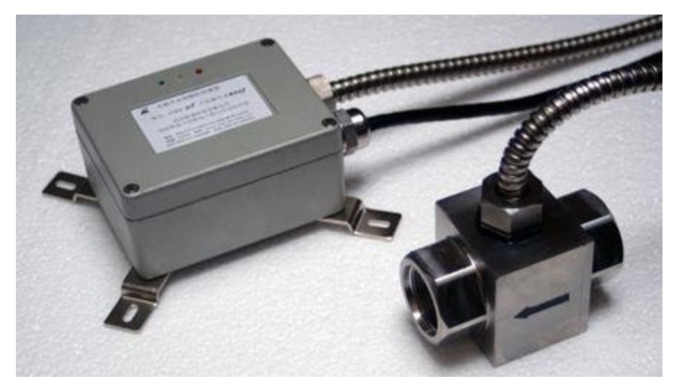
The ZCMS type of metal debris sensor.

Through comprehensive analysis, the oil multi-parameters that need to be monitored online include the density, temperature, kinematic viscosity, permittivity, and metal debris in the oil. In particular, kinematic viscosity and density reflect oil’s physical and chemical properties, while water content and permittivity can reflect indirectly the degree of oil pollution [27]. In addition, the metal debris was divided into three size ranges, namely small debris (with equivalent diameter no greater than 100 µm), medium debris (100–200 µm), and large debris (larger than 200 µm). The metal debris sensor is shown in Figure 3.

**Figure 4:**
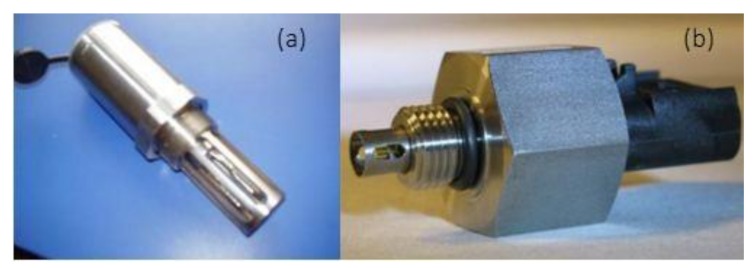
(**a**) The rate of water content sensor; (**b**) the oil quality sensor.

The ZCMS type of metal debris sensor was developed by ourselves, and can detect ferrous debris with an equivalent diameter of no less than 70 µm. Other indicators of oil were measured by the sensors from Shenzhen Front Wave Tech as shown in Figure 4.

The rate of water content can be measured by detecting the changing permittivity in the oil using the sensor shown in Figure 4a. The sensor in Figure 4b can measure the density, temperature, kinematic viscosity, and permittivity indicators, and is designed on the basis of the principle of component vibration. The fork is fixed at the natural resonance frequency. When the medium flows through the fork, the change in viscosity tends to change the value and amplitude of the resonant frequency, which can be used to judge the viscosity of the oil.

The above six oil parameters were combined as a set of sample sequences of the bearings data. After data preprocessing, the outcome, as the characteristic information of lubricating oil, was sent to G-KCM to classify the oil states. Bearing vibration signals can also be used for bearing wear states classification, which were handled by G-KCM, too. The results obtained from vibration signals were compared with those from the oil multi-parameters. The above two kinds of classification results were compared and analyzed.

**Figure 5:**
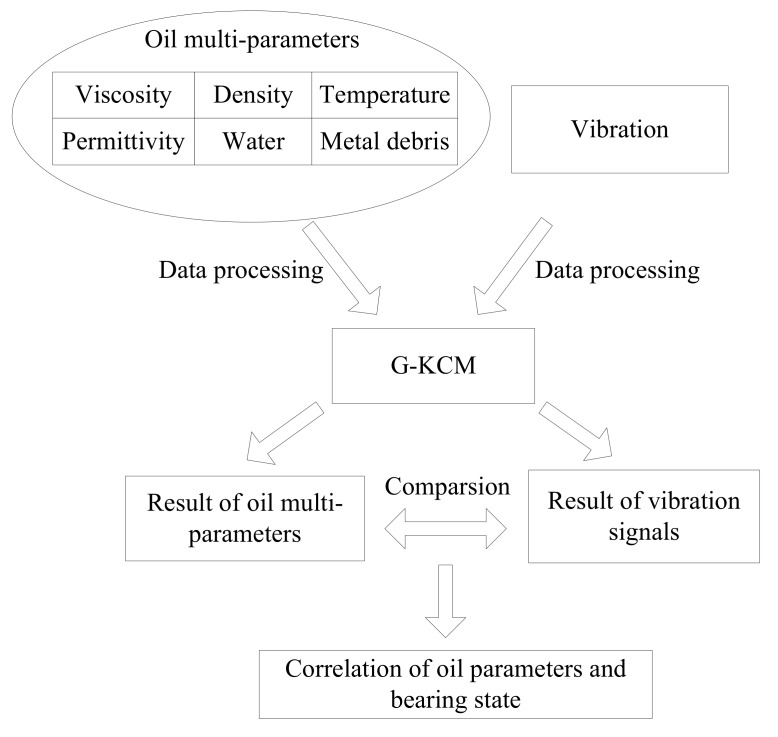
The implementation route of bearing states evaluation.

The implementation route of the bearing states evaluation is shown in Figure 5.

### 3.2. Experimental System and Platform Construction

**Figure 6:**
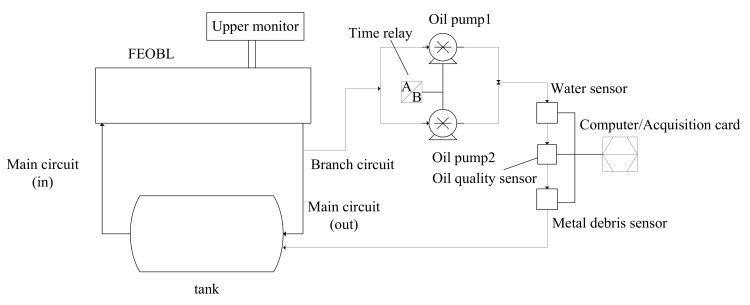
The block diagram of the FEOBL system.

The block diagram of the experimental system is shown in Figure 6.

**Figure 7:**
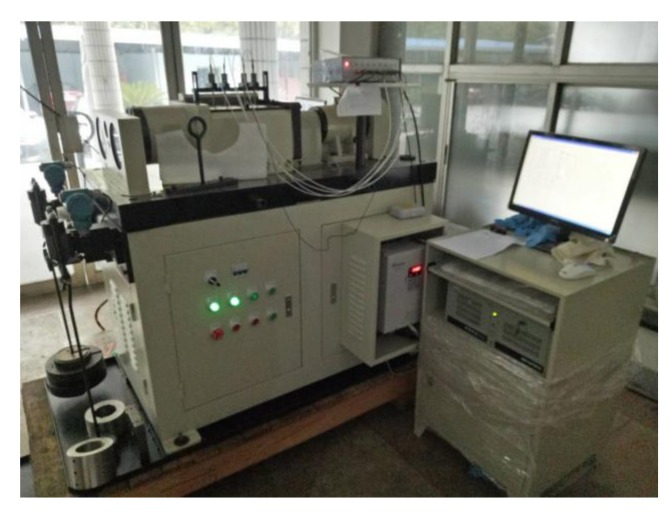
The ABLT-1A bearing fatigue testing machine.

The FEOBL system is mainly composed of the bearing fatigue testing machine (ABLT-1A) and the online oil multi-parameters monitoring system (OOMS). The ABLT-1A is manufactured by the company named HBRC (Hangzhou Bearing Test & Research Center), located in Hangzhou, Zhejiang province in china. The bearing fatigue testing machine is shown in Figure 7.

**Figure 8:**
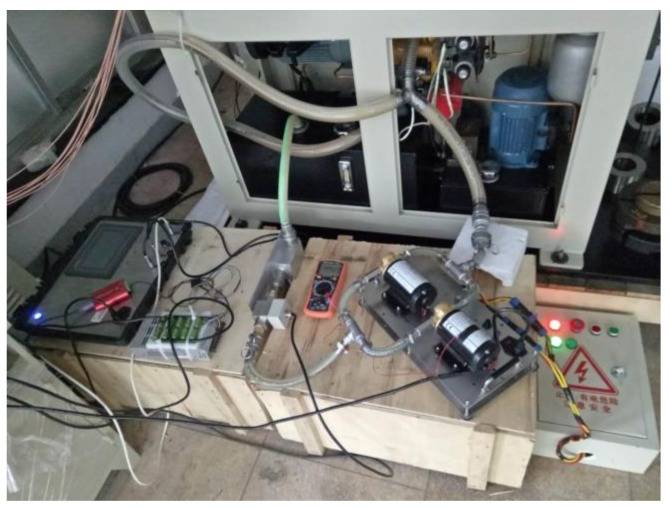
The OOMS and oil circulation loop.

The OOMS was integrated in the ABLT-1A to collect the data of metal debris, pollution, viscosity, and water, while various oil parameters and vibration signals were also recorded using a B&K4371 accelerator. The OOMS was constituted with a hardware system including oil multi-parameter monitoring sensors, signal conditioner, data acquisition device, and software system with functions including oil data display, recording, and storage. The vibration data acquisition card is ADLINK ACL-8112PG for IBM PC or compatible computers, which combines all data acquisition functions, such as A/D, D/A, DIO, and timer/counter into a single board. The ACL-8112PG card provides a high-speed sampling rate up to 100 kS/s at all gains, and features 16 single-ended inputs and bipolar inputs. In this test, the sampling rate is set at 20 kHz to acquire vibration signals. The OOMS and the oil circulation loop are shown in Figure 8.

## 4. Experiment Process and Results

### 4.1. Experiment Process

**Figure 9:**
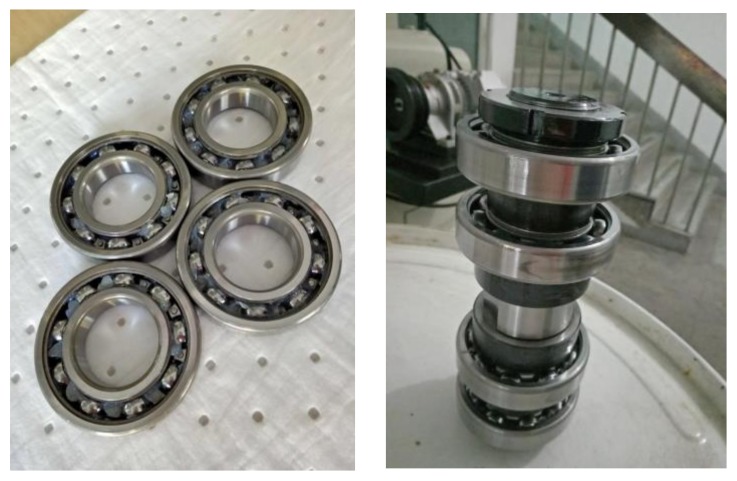
The assembled test bearing test setup.

The assembled test bearing test setup is shown in Figure 9. In the testing, a set of test bearings (four deep groove ball SKF6208 bearings) was installed on a rotating shaft which was lubricated by oil during the whole process.

The steps of experimental installation is as follows.

Step 1: Before installing the test head, select the large or small pistons according to the radial load of the test bearings.

Step 2: The inner of the bearings housing must be washed cleanly and free of stuff. The transmission spindle should be placed in the small hole on the right side of the test head with a small square shaft. Use the hand lever cam to retract it.

**Figure 10:**
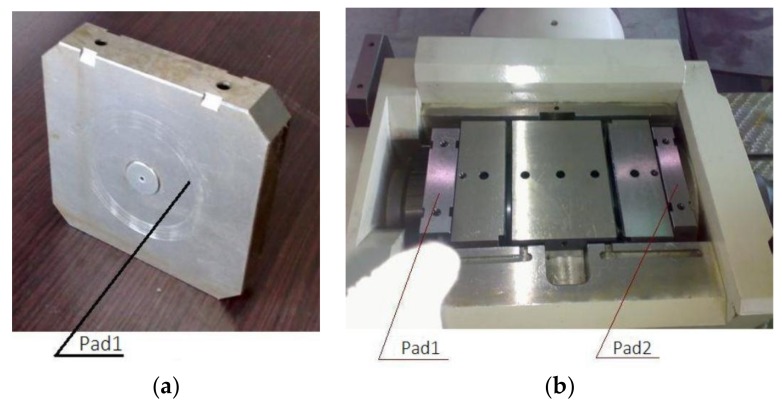
(**a**) The pad; (**b**) the fixed test bearings in the housing.

Step 3: Place the left and right pads in the housing to fix the test head as shown in Figure 10. Rotate the axial limit nut counterclockwise to the limit position, then rotate it clockwise 1/4 turn to loosen the two levers on both sides of the loading cylinder so that the piston can move forward freely.

**Figure 11:**
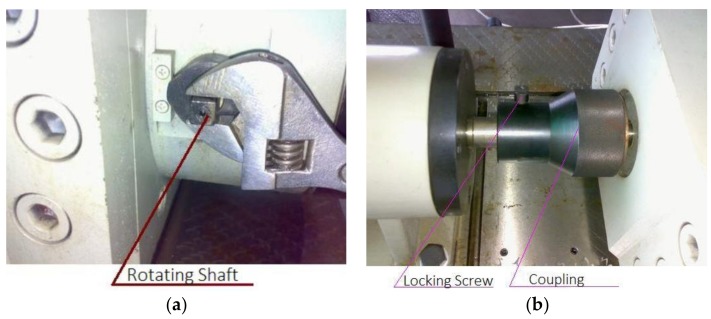
(**a**) One of the lever cams; (**b**) the assembled couplings.

Step 4: Connect the small square shaft and assemble the coupling as shown in Figure 11. Cover plate, and install temperature and vibration sensor.

At the beginning of the test, part of the data of the bearing grinding state is shown in Table 1 and Table 2.

During the experiment, in order to find a more reliable corresponding relationship between the oil monitoring parameters and bearing early wear characteristics in time, the test machine was stopped and the bearings had to be disassembled to inspect and search for signs of early wear failure of the bearings when the magnitude of bearing vibration signals increased to multiples of the initial stable value. Thus a 2 times root-mean-square value of initial vibration magnitude of the normal wear period had been set as the alarm threshold to shut down the test. Parts of the data of the bearings’ normal wear state during the test are shown in Table 3 and Table 4.

**Figure 12:**
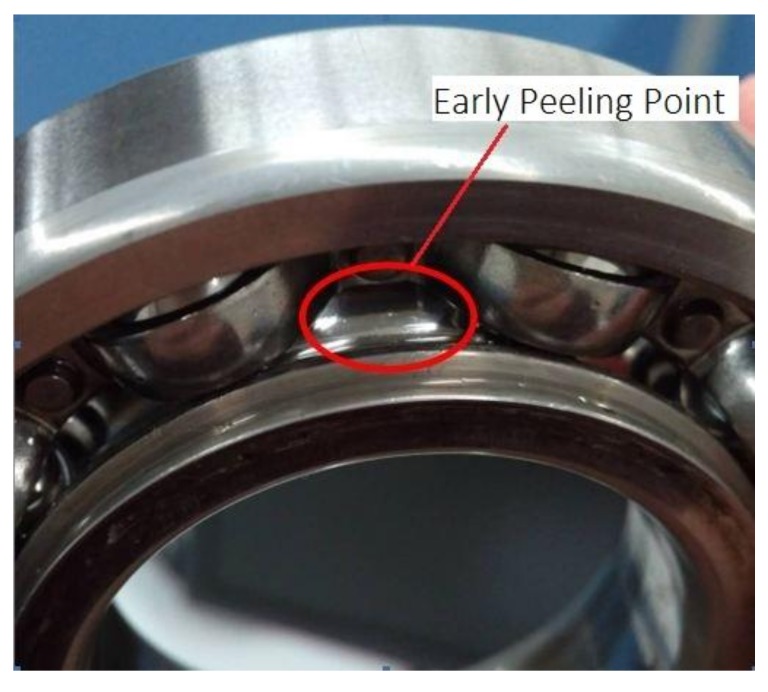
Early fatigue pitting failure in inner ring of bearing after running for 110 h.

During the test, the vibration level of the bearings raised to about 5 m/s^2^ after continuously working for 49 h under appropriate loads of about 24.4 MPa. Metal debris sensors had not detected metal debris with a diameter larger than 150 µm and bearings had entered the normal wear stage from the grinding stage. The vibration level of the bearings increased to 6 m/s^2^ after running about 110 h of the normal wear period. We stopped the machine and bearings were disassembled to check. We found that early fatigue pitting failure had occurred in the inner rings of the bearings as shown in Figure 12.

**Figure 13:**
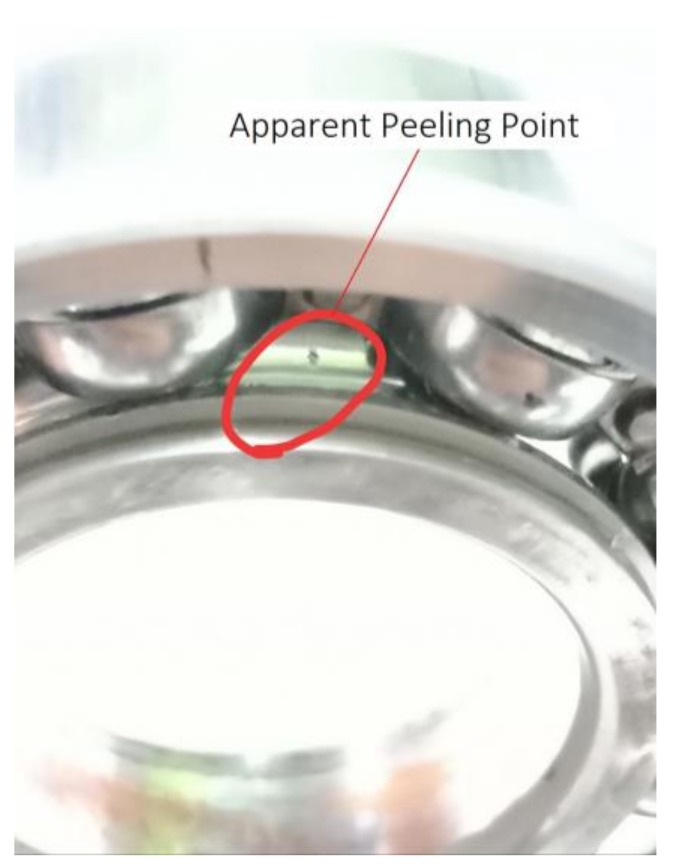
Peeling failure in inner ring of bearing after running for about 128 h.

**Figure 14:**
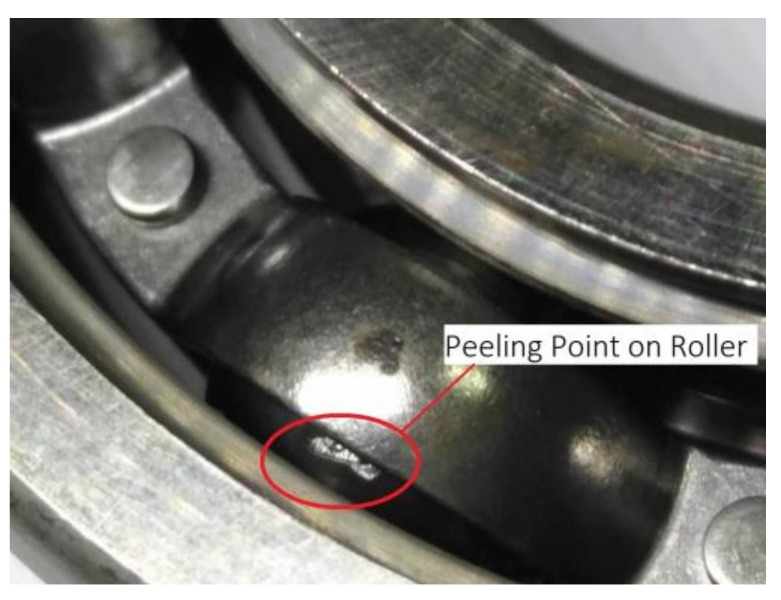
Peeling failure in one bearing ball after running for about 128 h.

The bearings were reassembled to continue testing, and afterwards, the machine stopped automatically when running to about 128 h. The recorded data showed that the vibration level had exceeded 8 m/s^2^ more than 50 times. We disassembled and checked the bearings again. There were clear peeling failures occurring on the surfaces of the inner ring and one rolling ball, which indicated that the bearing appeared to have peeling failure. The flaking failure of the bearing is shown in Figure 13 and Figure 14.

**Figure 15:**
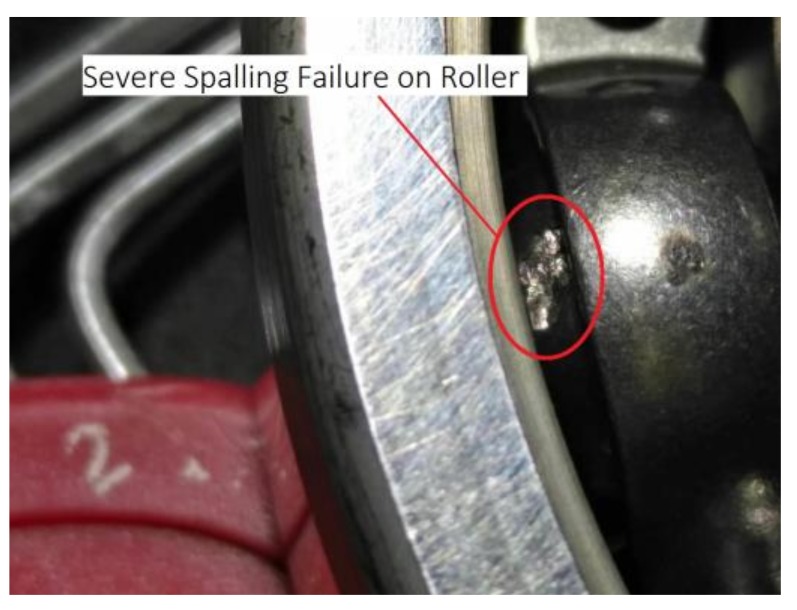
Severe peeling failure in one bearing ball after running for about 161 h.

In order to study further the change trends of oil composition during the whole lifetime, we raised the vibration threshold of stopping the test to 30 m/s^2^. Bearings were reassembled to continue the experiment. The machine stopped automatically again at about 161 h when the vibration level had exceeded the threshold more than 50 times. The online oil monitoring data showed that the metal debris increased significantly. After the bearings had been disassembled, we found that there was a large peeling point on the roller of the previous bearing as shown in Figure 15. The bearing had reached a severe failure.

Parts of the data of the bearings’ severe wear state during the test are shown in Table 5 and Table 6.

It can be seen from Table 1, Table 2, Table 3, Table 4, Table 5 and Table 6 that the different oil parameters have different sensitivities to bearing wear stage evolution in this experiment. The test had been carried out continuously so the moisture content was almost zero. With the increase of the number of metal debris per unit time, the oil permittivity kept increasing, though the increase quantity was relatively small. In addition, when the bearing failed, the small metal debris and medium metal debris had been detected, but large wear debris more than 200 µm had not been detected. Because of the low noise of the test environment, the noise-signal ratio of vibration signals was high and it had a high sensitivity, which was reliable as a control group to classify bearing wear states.

### 4.2. Evaluation of Bearing Wear States Based on Experimental Results

First we use KCM and G-KCM algorithms to cluster the online oil monitoring samples, respectively. We found that G-KCM is more effective than KCM. Then we used G-KCM to cluster bearing vibration signals and compared the clustering results with those of the online oil monitoring samples.

#### 4.2.1. Clustering Online Oil Monitoring Samples Based on KCM and G-KCM

It can be seen from Table 1, Table 3, and Table 5 that the parameter of moisture content is not worth analyzing and supposed to be excluded because it remains at a value of zero in the whole experiment. Due to most of the wear debris counts in the oil monitoring samples being also zeroes, an extremely weak white noise was added into the wear debris counts to avoid divergence while calculating the gray correlation matrix, which is the concrete preprocessing step that had been proposed in Section 2.2. Because the order of magnitude of density and permittivity are small, it was necessary to enlarge their difference to improve the resolution of these two parameters so as to avoid their data being overwhelmed by other parameters while calculating the gray correlation matrix.

**Figure 16:**
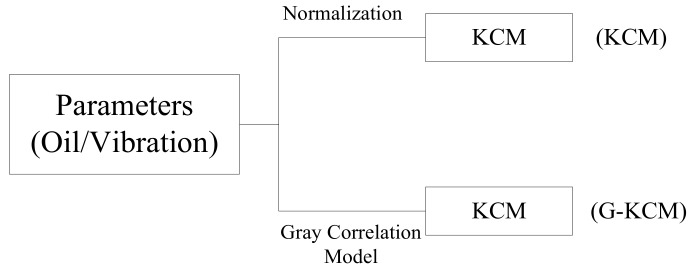
The specific calculation process of KCM and G-KCM.

The preprocessed data needs to be normalized prior to being analyzed directly using KCM as the control group. At the same time, the G-KCM algorithm was applied to the data without normalization as the test group. The specific calculation process is shown in Figure 16.

**Figure 17:**
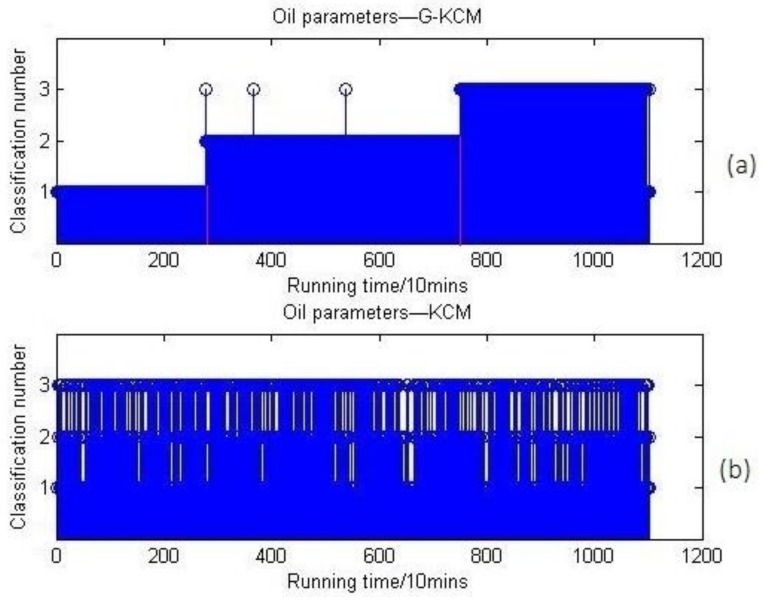
(**a**) Online oil multi-parameters clustering results based on G-KCM; (**b**) online oil multi-parameters clustering results based on KCM.

According to Equations (4) and (7), GCM has a function of dimensionless processing and distributing the data in (0, 1) the same as the normalization in order to make the calculation converge faster. In Section 2, Figure 1 makes it clear that the cluster number *k* should be set to 3, and the clustering results by the KCM and G-KCM algorithms are shown in Figure 17.

Figure 17a shows that when the preprocessing data were classified directly by KCM, the results are classified into chaos. In contrast, the analysis results by G-KCM are seemingly satisfactory. The ordinate represents the clusters of the bearing wear states. The abscissa represents the bearing testing time and the unit is 10 min. By means of G-KCM, this data have been clustered into three categories representing three stages of bearing wear states, respectively. The above comparison has seemingly shown that the improved G-KCM algorithm would be superior to the KCM method in clustering the online oil monitoring samples.

**Figure 18:**
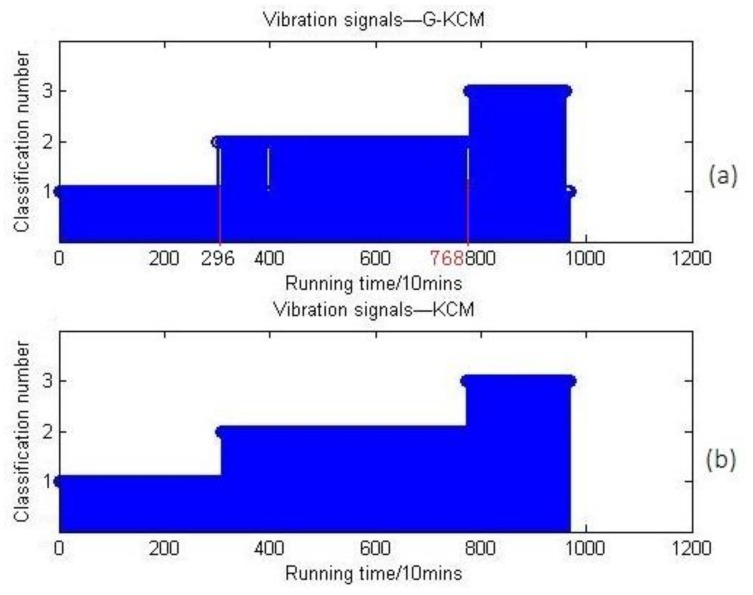
(**a**) Vibration signals clustering results based on G-KCM; (**b**) vibration signals clustering results based on KCM.

Although the comparison results have worked out, its reliability needs to be verified by further analysis. All vibration signals acquired in Section 4.1 are also analyzed using KCM and G-KCM. The results are shown in Figure 18.

Figure 18 shows that the vibration signals could be clustered into three categories whether or not G-KCM was used, because vibration signals have a high signal-to-noise ratio in the bearing test with a stable environment. That is to say, the clustering effect is acceptable whether or not vibration signals are pre-calculated by GCM. However, the fact is not as perfect as imagined. We find from Table 5 and Table 6 that the temperature parameter is a little bit abnormal. Generally speaking, the temperature of the machine for a longer run measurement should be higher, and both viscosity and density values depend on temperature. However, the tests were done in a non-stationary temperature situation, and the temperature of the machine cooled down absolutely for dozens of hours after the machine stopped automatically after running 128 h. Later, it restarted from 20 °C, and the temperature for the rest of the test was not over 56 °C anymore. Therefore, the above results are not convincing, and it is also necessary to analyze the clustering effects based on different parameter indicator combinations so as to figure out which indicators are more essential and valid, and how much weight the temperature occupies.

A variety of indicators of oil parameters were picked out and calculated by G-KCM and KCM. Supposing that all data could be entirely divided into three separate groups similar to those in Figure 1, and that the accuracy rate of the clustering result in Figure 17a was 100 percent, the relative values of the accuracy rate when using different multi-parameter combinations are presented in Table 7.

In Table 7, the indicators with the symbol “√” are chosen as inputs in the clustering algorithm. Table 7 shows that all combinations of different indexes without temperature are not better than those that include the temperature index, which means the temperature parameter dominates the results of G-KCM in Figure 17a. Besides, what is more important is that the more oil parameter combination in the analysis, the higher the accuracy rate of G-KCM. This is because the more indicators that are used, the more comprehensive the information is. As for KCM, its accuracy rate is greatly influenced by the data of temperature and metal debris whose magnitude is ten times smaller than the temperature. We can find that KCM is dominated by temperature and regards the minimal values like metal debris as a noise that disturbs clustering results, although the metal debris parameter is a greatly important reference index for evaluating the wear state of bearings. To sum up, for oil parameters, it is necessary to analyze multiple parameters for evaluating bearing status, and also, G-KCM is more reliable than KCM.

**Figure 19:**
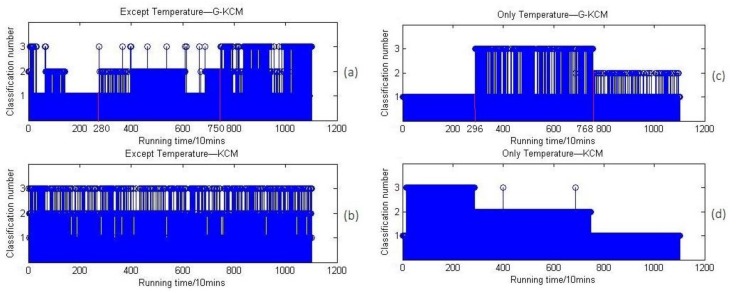
(**a**) Oil parameters except temperature clustering results based on G-KCM; (**b**) oil parameters except temperature clustering results based on KCM; (**c**) only temperature clustering results based on G-KCM; (**d**) only temperature clustering results based on KCM.

The oil parameters except the temperature index and only the temperature index are recalculated, respectively, and the results are shown in Figure 19.

Also, Figure 19c,d shows that the temperature has played the most important role in the above clustering results. The third edge of stage at the 7680 min point is probably because the machine became colder when it shut down automatically. However, at about the 2960 min point, the temperature increased steadily and consecutively and this trend of increasing remained stable within 128 h. Then in Figure 19a, the changes of stages at about 2800 min and 7500 min are due to two possible causes: the first one could be that the three separate groups were divided only because of a non-stationary temperature situation, and the other one could be that the oil parameters under a stationary temperature situation were able to be clustered as three similar stages as in Figure 1, which is just covered and disturbed by the factor of temperature like Figure 17a.

**Figure 20:**
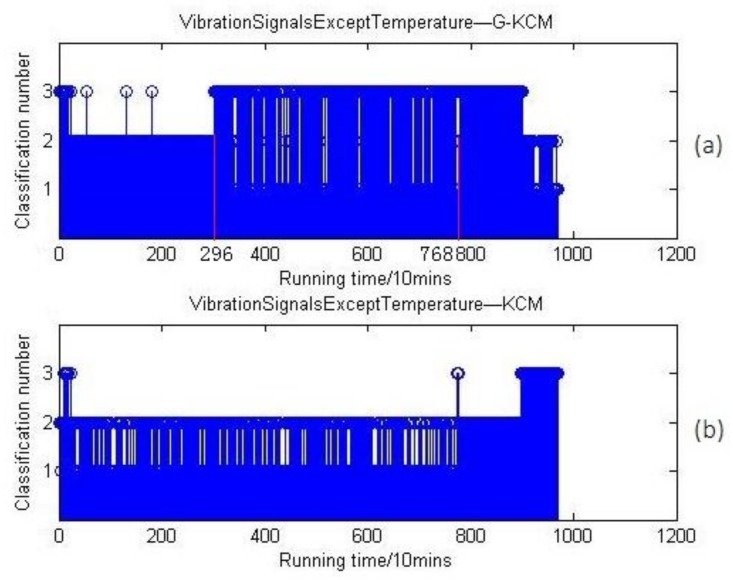
(**a**) Vibration signals except temperature clustering results based on G-KCM; (**b**) vibration signals except temperature clustering results based on KCM.

To further verify the effect of temperature on the results, the parameters of vibration signals except temperature index were recalculated, and the results are shown in Figure 20.

In general, bearing vibrations are hardly affected by temperature. In this test, the RMS values of vibration signals continuously increased in whole-life testing of the bearings. Figure 20a shows that the first change of stage at the 2960 min point was found out by G-KCM, which is similar with Figure 19a, and the second state can be reflected by the beating characteristics of classification number from 2960 min to 7680 min, which illustrates that the vibrating value of bearings in normal wear stage fluctuate widely because of extension at tiny pitting failures. It is also easy to find out the second state of bearings in Figure 20b. To sum up, vibration signals can reflect the wear states of bearings effectively.

**Figure 21:**
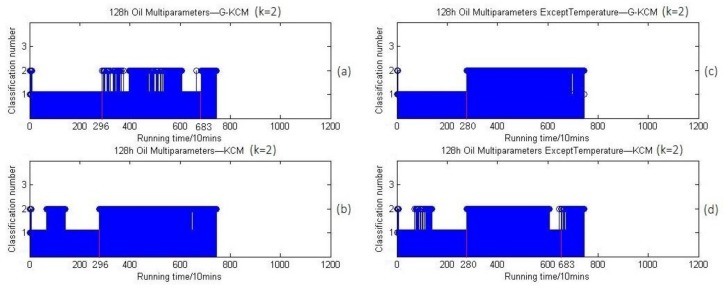
(**a**) 2-Clustering results of oil parameters within 128 h based on G-KCM; (**b**) 2-Clustering results of oil parameters within 128 h based on KCM; (**c**) 2-Clustering results of oil parameters except temperature within 128 h based on G-KCM; (**d**) 2-Clustering results of oil parameters except temperature within 128 h based on KCM.

For further verification that the oil parameters have the ability to reflect the wear states of bearings truly and effectively in the stationary temperature situation, all data from 7680 min to the end, while the temperature was not over 56 °C, were deleted. At the same time, the temperature from 2800 min to 3000 min was consecutive, increasing continuously. Suppose that the deleted part is the third stage of bearing wear state, severe wear, then the rest of the data is supposed to be divided into two groups. To exclude the effect of classification quantity on clustering results, the k of the clustering algorithm was set as two and three, respectively, to cluster the oil parameters. When k=2, the 2-clustering results of the oil parameters and that excluding temperature within 128 h are shown in Figure 21.

**Figure 22:**
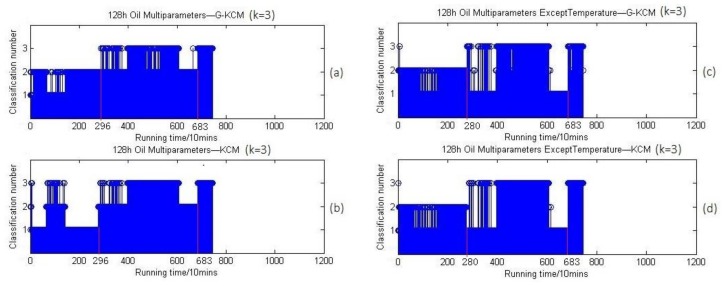
(**a**) 3-Clustering results of oil parameters within 128 h based on G-KCM; (**b**) 3-Clustering results of oil parameters within 128 h based on KCM; (**c**) 3-Clustering results of oil parameters except temperature within 128 h based on G-KCM; (**d**) 3-Clustering results of oil parameters except temperature within 128 h based on KCM.

And when k=3, the 3-clustering results are shown in Figure 22.

Figure 21 and Figure 22 show that clustering of the oil parameters in a stationary temperature situation can be done similarly in two separate groups whether *k* is two or three, and the first change of stage is at the 2800 min point approximately similar to the results of vibration signals except temperature in Figure 20a. More importantly, Figure 21c,d show that the first time points of clusters conversion is 2800 min, which is the same as Figure 22c,d, while the counterpart in Figure 21a,b, as well as in Figure 22a,b, is 2960 min. This result means the clustering of the temperature that includes its trend of changing is independent and a little different from other oil parameters, which exactly verifies the second possible cause that the oil parameters under a stationary temperature situation can be clustered as three similar stages as in Figure 1, which is just covered and disturbed by the factor of temperature. In summary, the above comparisons indicate that the evaluation approach for bearing wear states based on oil multi-parameters is feasible.

To explore the applicability, merits, and defects of the proposed model under certain conditions, it is compared with other models that are usually used to analyze for evaluating bearing health conditions. The comparison of performance of each model is shown in Table 8.

Table 8 shows that it is an important method to analyze the bearing status based on vibration signals such as EEMD (Ensemble Empirical Mode Decomposition), and G-KCM is feasible to assess the bearing wear states in the case that multiple oil parameters would be accessible in a gray hydraulic lubrication system.

#### 4.2.2. Comparison of Clustering Results Based on Oil Multi-Parameters and on Vibration Signals by G-KCM Algorithm

By means of the G-KCM algorithm, the oil multi-parameters and vibration signals of bearings through their whole lifetime were calculated and compared. The analysis based on oil parameters was set as a new test group and the vibration signals analysis as a new control group. It was possible to find the difference by comparative analysis.

By comparing Figure 20a with Figure 22c, we find that the clustering results with the oil multi-parameters are nearly consistent with that of vibration signals, and Figure 22c shows that the first time point of clustering conversion (wear stage conversion) in the horizontal coordinates was 2800 min (46.6 h) while that point in Figure 20a was at 2960 min (49.3 h). According to the experimental process log file and records of OOMS, the first time for the FEOBL to stop automatically when running was 49 h, and the second time was 128 h because the vibration signal magnitude had exceeded the predefined threshold. The time for bearing wear stages conversion was basically consistent with the results based on oil multi-parameters and vibration signals by the G-KCM algorithm. This means the lubricating oil multi-parameters contain vital information that can be used to find the approximate time points when the bearing wear state is to change. The reliable correlation between the online oil parameters and bearing wear state has been established. At the same time, it should be noted that a clustering conversion at 6830 min (113.8 h) appears in Figure 19a, Figure 21, and Figure 22, which does not depend on temperature. The fluctuation of data between clusters before the third stage means the advancing trend of oil parameters changes or fluctuates prior to the severe wear state of bearings. It is not accidental and this term will be discussed together with the problem why the wear stage conversion time points in the clustering results based on oil parameters were earlier than that based on vibration signals in Section 5.

## 5. Discussion

It can be seen from the comparison between Figure 20 and Figure 22c that the wear stage conversion time points based on oil multi-parameter analysis are a few hours earlier than that based on vibration signals analysis. It reveals that oil parameters contain earlier fault warning information than vibration signals, and oil information is more suitable for early fault prognostics. The reason is that what vibration signals reflect is the bearing vibration level in real time, and only when there is a failure already existing the vibration magnitude changes abruptly. The information in lubricating oil reflects the effect of changing bearing wear state on the physical and chemical properties of lubricating oil during operation, such as infiltrating impurities into oil or altering its chemical composition. These changes are recorded continuously and reflected by various oil indexes. When the bearing is about to fail or has an early failure, it has been recorded into the oil information in real time and quickly, which can be presented through mining and analyzing the online oil monitoring data. This explains the phenomenon that fluctuation of data category appears invariably before the third stage, with the incomplete consistency of the time points in the comparison results. It is not easy for the online monitoring method based on vibration signals to achieve accurate prediction of early wear failure. Online oil multiple-parameters have more potential in bearing early fault prognostics than vibration signals.

## 6. Conclusions

In this paper, we built up the fatigue experiment system for bearing whole-lifetime testing and online oil multi-parameter monitoring systems. The G-KCM clustering method based on the k-means clustering model optimized by a gray correlation model was proposed. Then it was used to cluster the bearings oil data and vibration signals. As a result, the reliable correlation between lubricating oil and bearing wear states has been established, since the time for bearing wear stages conversion is basically consistent with the results based on oil multi-parameters and vibration signals by the G-KCM algorithm. Also, analysis results show that there is an obvious correspondence between the clusters and the bearing wear states that the lubricating oil multi-parameters contain vital information that can be used to find the approximate time points when the bearing wear state is to change. The comparison of the clustering results based on oil multi-parameters and on vibration signals has shown that online oil data have early wear failure prediction ability for bearings superior to vibration signals. It is expected to realize online oil monitoring and evaluation for bearing health conditions and to provide an approach for early identification of bearing-related failure modes. Further research is to apply this model to the prediction of bearing residual life based on the online oil multi-parameters.

## Figures and Tables

**Table 1:** Part of online oil monitoring data during grinding of bearings.

Viscosity (cp)	Density (g/cc)	Water (%)	Permittivity (F/m)	Temperature (°C)	Granule	Mid-Particle
25.22	0.87	0	2.27	52.13	0	0
26.95	0.87	0	2.27	52.22	0	0
22.42	0.87	0	2.27	52.25	0	0
28.22	0.86	0	2.26	52.28	0	0
39.0.9	0.84	0	2.27	52.31	0	0
25.38	0.87	0	2.27	52.41	0	0
23.98	0.85	0	2.27	52.38	0	0
26.36	0.86	0	2.26	52.41	0	0
26.64	0.86	0	2.26	52.41	0	0
30.58	0.86	0	2.26	52.41	0	0

**Table 2:** Part data of the test machine status during grinding of bearings.

Temperature 1 (°C)	Temperature 2 (°C)	Temperature 3 (°C)	Temperature 4 (°C)	Radial Load (MPa)	Axial Load (MPa)	Speed (r/s)	RMS Value (m/s^2^)	Peak Factor	Kurtosis
50.3	50.9	50.2	50.4	24.3	0.4	3979	3	3.1	2.9
51.8	52.2	51.7	51.9	24.3	0.4	3981	3	3.1	3
53	53.4	53	53	24.4	0.4	3981	3.2	3.3	3.1
54	54.9	54	54.1	24.4	0.4	3988	3.5	3.2	2.8
54.8	55.3	54.8	54.7	24.4	0.4	3981	3.9	5.6	5.9
55.6	56.2	55.5	55.5	24.3	0.4	3988	4	3.5	3.1
56.2	56.8	56.1	56	24.3	0.4	3994	3.8	3.6	3.2
56.7	57.1	56.7	56.9	24.4	0.4	3986	4	3.7	3.2
57.3	57.7	57.1	57.2	24.3	0.4	3996	3.8	3.5	3.2
57.8	58.3	57.5	57.6	24.4	0.4	3988	3.9	3.2	3.1

**Table 3:** Part of online oil monitoring data during normal wear of bearings.

Viscosity (cp)	Density (g/cc)	Water (%)	Permittivity (F/m)	Temperature (°C)	Granule	Mid-Particle
55.4	0.77	0	2.25	62.47	0	0
47.38	0.79	0	2.26	62.44	0	0
58.23	0.8	0	2.26	62.47	0	0
90.89	0.68	0	2.25	62.50	0	0
57.23	0.76	0	2.25	62.41	0	0
51.55	0.75	0	2.25	62.50	0	0
91.25	0.67	0	2.25	62.47	0	0
54.17	0.77	0	2.25	62.50	0	0
76.56	0.66	0	2.25	62.47	0	0
48	0.78	0	2.26	62.44	0	0

**Table 4:** Part data of the test machine status during normal wear of bearings.

Temperature 1 (°C)	Temperature 2 (°C)	Temperature 3 (°C)	Temperature 4 (°C)	Radial Load (MPa)	Axial Load (MPa)	Speed (r/s)	RMS Value (m/s^2^)	Peak Factor	Kurtosis
64.5	64.4	64	63.9	24.4	0.4	3973	5.8	3.5	3
64.6	64.2	64	64	24.4	0.4	3973	5.8	3.6	3.3
64.6	64.4	64	64	24.4	0.4	3971	5.6	2.9	2.6
64.7	64.6	64.1	63.9	24.4	0.4	3988	5.8	2.9	2.8
64.6	64.1	64.1	64	24.4	0.5	3981	5.1	2.8	2.6
64.7	64.6	64.2	64.1	24.4	0.4	3981	5.6	3.7	3.2
64.7	64.4	64.2	64	24.4	0.4	3981	5.9	3.3	2.9
64.8	64.6	64.2	64.1	24.4	0.4	3973	5.1	3.3	3
64.8	64.6	64.2	64.1	24.4	0.4	3973	5.6	3.3	3
64.9	64.8	64.3	64.2	24.4	0.4	3979	5.8	3.2	3

**Table 5:** Part of online oil monitoring data during severe wear of bearings.

Viscosity (cp)	Density (g/cc)	Water (%)	Permittivity (F/m)	Temperature (°C)	Granule	Mid-Particle	Large Particle
38.22	0.88	0	2.28	50.06	0	0	0
37.80	0.87	0	2.28	50.03	2	0	0
34.64	0.87	0	2.28	50.06	0	0	0
29.70	0.87	0	2.28	50	1	0	0
35.30	0.87	0	2.28	49.97	0	0	0
41.81	0.85	0	2.28	50.06	0	0	0
56.61	0.83	0	2.28	50.03	0	0	0
42.86	0.83	0	2.28	50.03	1	0	0
43.52	0.85	0	2.28	50.09	0	0	0
36.11	0.86	0	2.28	50.03	0	0	0

**Table 6:** Partial data of the test machine status during serious wear of bearings.

Temperature 1 (°C)	Temperature 2 (°C)	Temperature 3 (°C)	Temperature 4 (°C)	Radial Load (MPa)	Axial Load (MPa)	Speed (r/s)	RMS Value (m/s^2^)	Peak Factor (m/s^2^)	Kurtosis
50.3	51.6	51.4	51.1	14.6	0.4	4032	11.6	9.4	24.9
50.3	52.1	51.4	51	14.6	0.4	4031	5.8	5.7	6.6
50.3	51.8	51.4	51.1	14.6	0.4	4025	9	6.6	10.5
50.3	51.9	51.4	51.1	14.6	0.4	4038	15.2	7.3	19.3
50.3	52	51.5	51.1	14.6	0.4	4032	16.7	7	15.1
50.3	51.9	51.5	51.1	14.6	0.4	4032	18.2	6.5	15.2
50.3	52	51.5	51.1	14.6	0.4	4031	11.3	7.9	19.6
50.3	52	51.5	51.2	14.6	0.4	4032	13.7	6.3	16.4
50.3	52	51.5	51.2	14.6	0.4	4032	7.8	6.1	10.4
50.3	52	51.5	51	14.6	0.4	4032	11.2	7.6	16.9

**Table 7:** The accuracy rate of different multi-parameter combinations.

Viscosity (cp)	Density (g/cc)	Permittivity (F/m)	Temperature (°C)	Granule	Mid-Particle	Accuracy Rate of G-KCM (%)	Accuracy Rate of KCM (%)
			√			94.11	99.60
√	√					58.35	82.65
√		√				55.54	80.24
√			√			66.97	88.29
	√	√				35.18	55.18
	√		√			31.12	99.59
		√	√			67.42	99.59
√	√	√				82.21	20.80
√	√		√			97.19	92.47
√		√	√			89.66	92.29
	√	√	√			96.37	99.55
√	√	√	√			97.64	92.47
√				√	√	66.61	35.39
	√			√	√	35.21	35.57
		√		√	√	48.64	35.57
			√	√	√	36.93	35.57
√	√			√	√	85.21	35.39
√		√		√	√	51.85	35.39
√			√	√	√	50.88	36.30
	√	√		√	√	66.97	35.57
	√		√	√	√	91.56	35.57
		√	√	√	√	90.19	35.57
√	√	√		√	√	82.12	35.39
√	√		√	√	√	95.92	36.30
√		√	√	√	√	92.68	36.30
	√	√	√	√	√	95.19	35.57
√	√	√	√	√	√	95.92	36.30

**Table 8:** The comparison of performance of each model.

Method	Parameter	Defect	Benefits
EMD	Vibration	Frequent appearance of modal aliasing	Well done in non-linear non-stationary signals
EEMD	Vibration	Tedious process	High accuracy
PCA	Vibration	Undefined by negative factors	Dimension reduction
ICA	Vibration	The unsteady least squares method	Simplify, Explainable
BPNN	Vibration /Oil	Local optimum solution	Grey system
KCM	Vibration /Oil	Local optimum solution	Simple algorithm
G-KCM	Vibration /Oil	Inaccuracy with few parameters	Grey system, High signal to noise ratio
